# Supplementary material for: SuPAR mediates viral response proteinuria by rapidly changing podocyte function
Source: Nat Commun. 2023 Jul 21;14:4414. doi: 10.1038/s41467-023-40165-5 (PMC10362037; doi:10.1038/s41467-023-40165-5)

Supplementary Information

SuPAR mediates viral response proteinuria by rapidly changing podocyte function

Changli Wei[1], Prasun K. Datta[2], Florian Siegerist[3,4], Jing Li[1], Sudhini Yashwanth[1], Kwi Hye Koh[5], Nicholas W Kriho[6], Anis Ismail[7], Shengyuan Luo[1], Tracy Fischer[2], Kyle T Amber[1,8], David Cimbaluk[6], Alan Landay[1], Nicole Endlich[3,4], Jay Rappaport[2], Michigan Medicine COVID-19 Investigators*, Salim S Hayek[7], Jochen Reiser[1]

[1] Department of Medicine, Rush University Medical Center, Chicago, IL, USA

[2] Tulane National Primate Research Center, Covington, LA 70433, USA

[3] Department of Anatomy and Cell Biology, University Medicine Greifswald, 17487 Greifswald, Germany

[4] NIPOKA GmbH, 17489 Greifswald, Germany.

[5] Morphic Therapeutic, Waltham, MA 02451, USA

[6] Department of Pathology, Rush University Medical Center, Chicago, IL, USA

[7] Division of Cardiology, Department of Internal Medicine, University of Michigan, Ann Arbor, MI, USA

[8] Department of Dermatology, Rush University Medical Center, Chicago, IL, USA

Corresponding authors:

Changli Wei, MD, PhD: changli_wei@rush.edu

Salim S Hayek, MD: shayek@med.umich.edu

Jochen Reiser MD, PhD: jochen_reiser@rush.edu

* List of the Michigan Medicine COVID-19 (M^2C^2) Investigators:

Alexi Vasbinder[7], Anis Ismail[7], Elizabeth Anderson[7], Tonimarie Catalan[7], Ian Pizzo[7], Brayden Bitterman[7], Grace Erne[7], Kristen Machado-Diaz[7], Feriel Presswalla[7], Namratha Nelapudi[7], Kingsley-Michael Amadi[7], Alina Bardwell[7], Pennelope Blakely[7], Yiyuan Huang[7], Mousumi Banerjee[7], Rodica Pop-Busui[7], Salim S. Hayek[7]

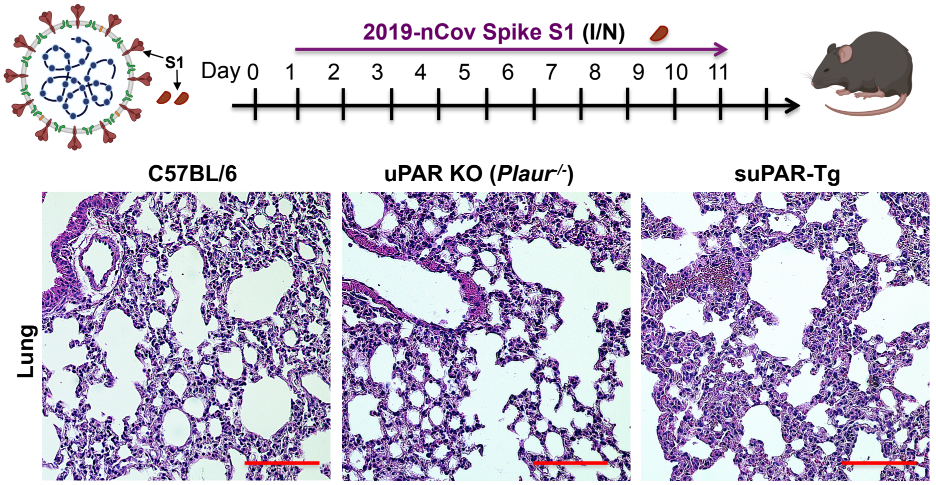

Supplementary Fig 1. Lung histopathology in 2019-nCov spike S1 protein inoculated mice. As shown in the scheme, 2019-nCov spike S1 protein (2.5 ng/g body weight) was inoculated intranasally (I/N) once a day for 10 days. Mice (n=4 per group) were sacrificed on day 11 for lung histopathological examination. Periodic acid-Schiff (PAS) staining revealed various degrees of injuries including septal thickening, neutrophil infiltration, edema and focal hemorrhage in lung tissues of all three tested strains of mice after 2019-nCov spike S1 treatment. uPAR-KO, urokinase receptor knockout mice; suPAR-Tg, mouse soluble urokinase receptor transgenic mice. Red scale bar, 100 μm. The experiments were repeated 4 times, with 4 similar images obtained each time in each group. Illustrations of SARS-Cov-2 and mouse were created with BioRender.com.

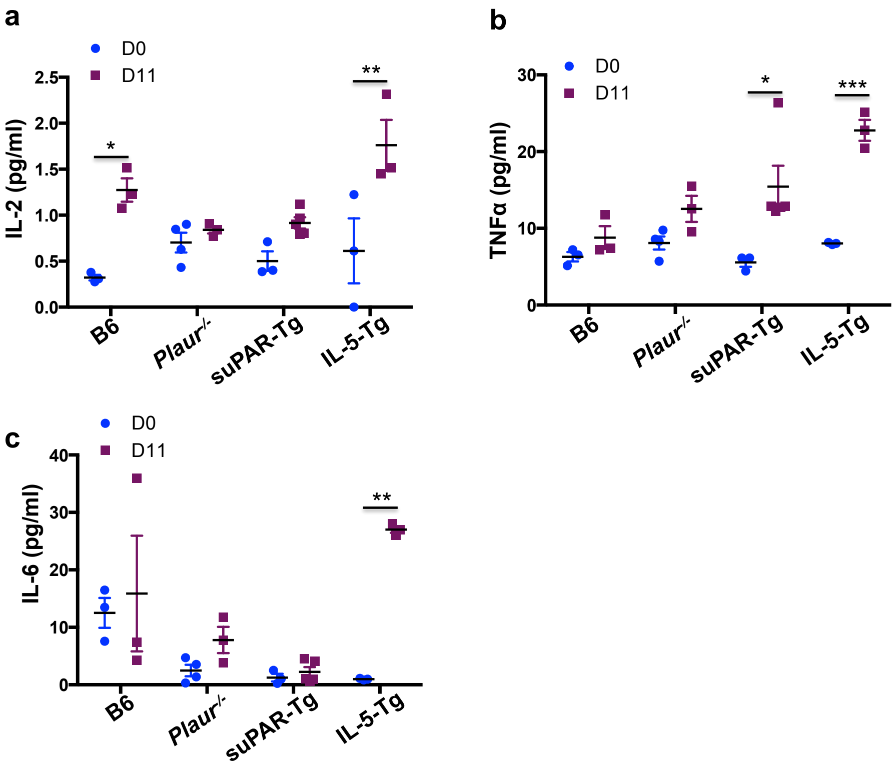

Supplementary Fig 2. Elevation of circulating cytokines in 2019-nCov S1 protein inhaled mice. Circulating cytokines were measured with proinflammatory panel 1 (mouse) kits before (D0) and after 2019-nCov S1 protein inoculation (D11). n=3 biological replicates for B6 and IL-5-Tg, n=4 for *Plaur*$^{-/-}$, n=5 for suPAR-Tg (some serum samples were not available for measurement). Among assayed cytokines, IL-2 (**a**), TNFα (**b**) and IL-6 (**c**) levels were increased in different tested strains of mice after inoculation of 2019-nCov S1 protein (D11), compared to baseline levels (D0). Data were presented as mean ± SEM. * P<0.05, ** P<0.01, *** P<0.001. Two-way ANOVA with Tukey's multiple comparisons test.

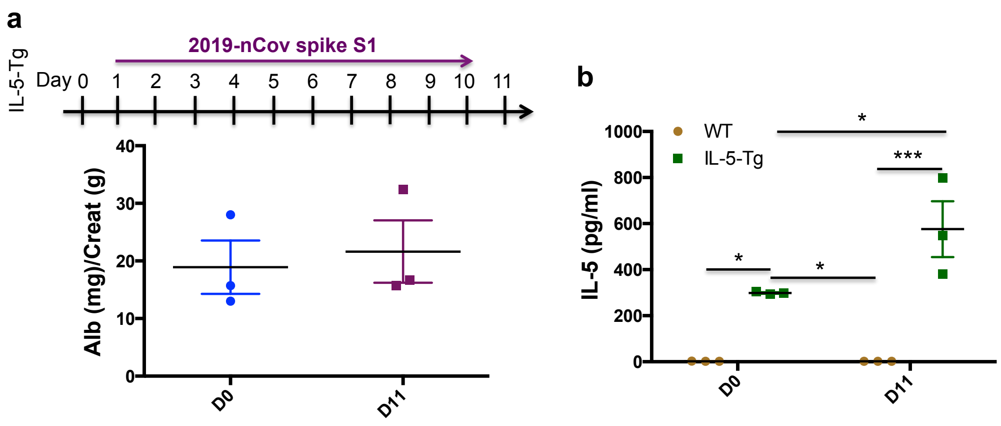

Supplementary Fig 3. Inoculation of 2019-nCov S1 protein did not cause proteinuria in mice with high levels of IL-5. a Experimental scheme and proteinuria profiles before (D0) and after 2019-nCov S1 protein inoculation (D11). n=3 mice. Alb, albumin; Creat, creatinine. Data were shown as mean ± SEM. Two-tailed *t*-test, *P*=0.723, non-significant. **b** Circulating IL-5 levels before (D0) and after 2019-nCov S1 protein inoculation (D11). n=3 mice per group. Data were presented as mean ± SEM. * *P*<0.05, *** *P*<0.001. Two-way ANOVA with Tukey's multiple comparisons test.

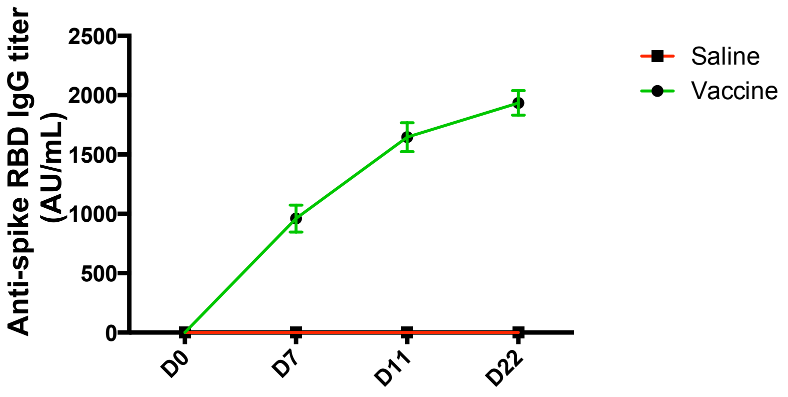

Supplementary Fig 4. Circulating anti-spike RBD IgG titers in BNT162b2 vaccinated suPAR-Tg mice. Age matched male suPAR-Tg mice were randomly selected to receive single dose of SARS-Cov-2 vaccine BNT162b2 or same amount of phosphate buffered saline intramuscularly (n=6 per group). Serum was collected before (D0) and different days after administration of SARS-Cov-2 vaccine BNT162b2 or saline. Anti-spike RBD antibody titers were assayed with GENLISA mouse anti-SARS-Cov-2 IgG antibody to spike RBD protein quantitative titration ELISA kit. AU, artificial unit; D, day. Data were presented as mean ± SEM.

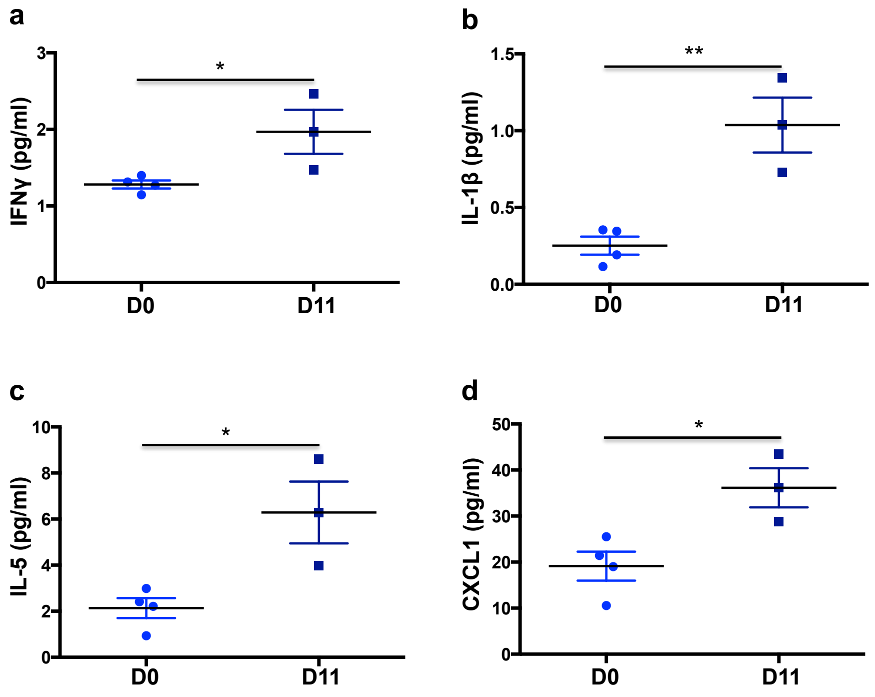

Supplementary Fig 5. Inoculation of 2019-nCov nucleocapsid protein into suPAR-Tg mice elicited elevation of cytokines in blood circulation. Age matched male suPAR-Tg mice were randomly selected to inoculate with 2019-nCov nucleocapsid protein once a day for 10 days (n=4). Serum was collected before (D0) and after last dose of nucleocapsid (D11) to measure proinflammatory cytokines with multi-spot assay system, proinflammatory panel 1 (mouse) kit from MSD (1 sample at D11 was not available for measurement). P=0.0392 for IFNγ (**a**), P=0.005 for IL-1β (**b**), P=0.02 for IL-5 (**c**), P=0.0216 for CXCL1 (**d**). Data were presented as mean ± SEM. * P<0.05, ** P <0.01. two-tailed Student's *t*-test (**a**-**d**).

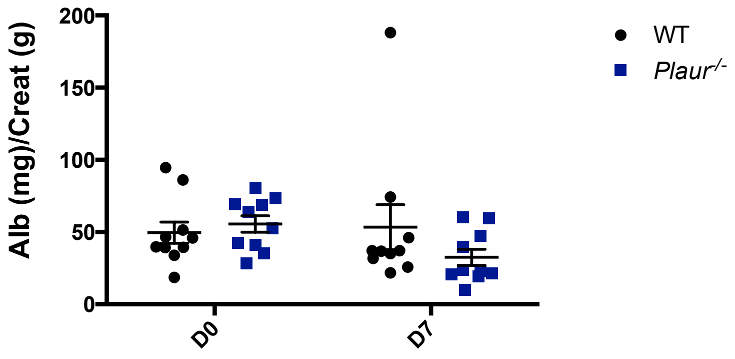

Supplementary Fig 6. Injection of HIV-1 gp120 protein into wild type C57BL/6j or *Plaur*$^{-/-}$ mice did not cause proteinuria. Age and sex matched C57BL/6j or *Plaur*$^{-/-}$ mice (n=10 per group, 5 male, 5 female) were administered with HIV-1 gp120 intraperitoneally in the same scheme as with suPAR-Tg mice. Urine was collected before (D0) and after (D7) last dose of HIV-1 gp120 to profile proteinuria. Alb, albumin; Creat, creatinine. Data were shown as mean ± SEM. Two-way ANOVA with Tukey's multiple comparisons test. Not-significant.

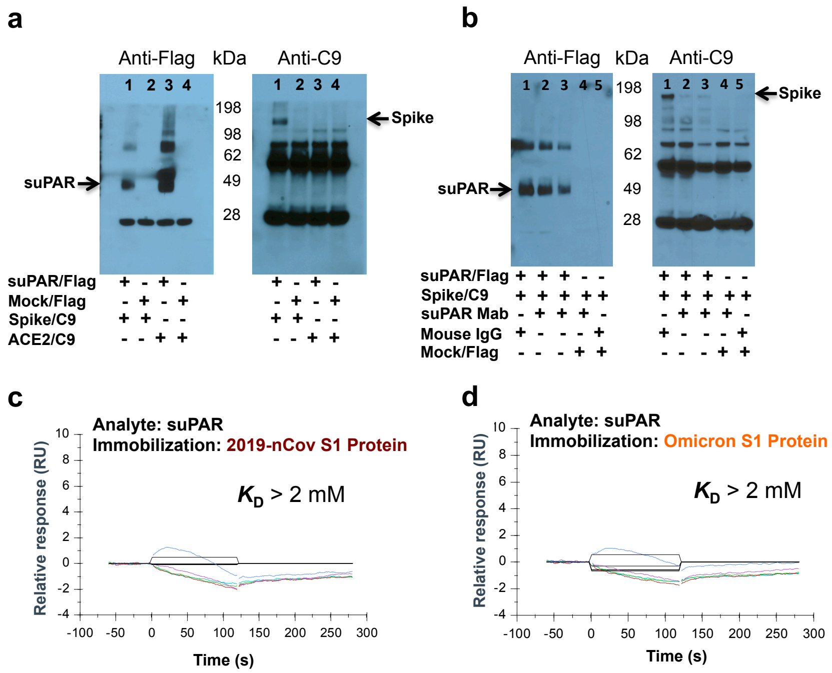

Supplementary Fig 7. Assay dependent suPAR interactions with spike S1 protein. a, **b** Plasmid DNA encoding SARS-CoV-2 spike protein, human suPAR isoform 1, human ACE2 respectively were transfected into HEK293 cells. Co-immunoprecipitation assays were performed with antibodies against the corresponding tag (C9 for spike and ACE2; Flag for suPAR). The experiments were repeated twice, giving similar results. Molecular marker (kDa) was provided in the middle between 2 blots. **a** suggests the interaction between spike protein and suPAR (Lane 1), but not between ACE2 and suPAR (Lane 3). **b** shows uPAR antibody could block the interaction between spike protein and suPAR (Lane 2, 3). Mab, monoclonal antibody. **c**, **d** Surface plasmon resonance assays indicate that neither 2019-nCov S1 (**c**) nor its Omicron counterpart (**d**) bound suPAR well.

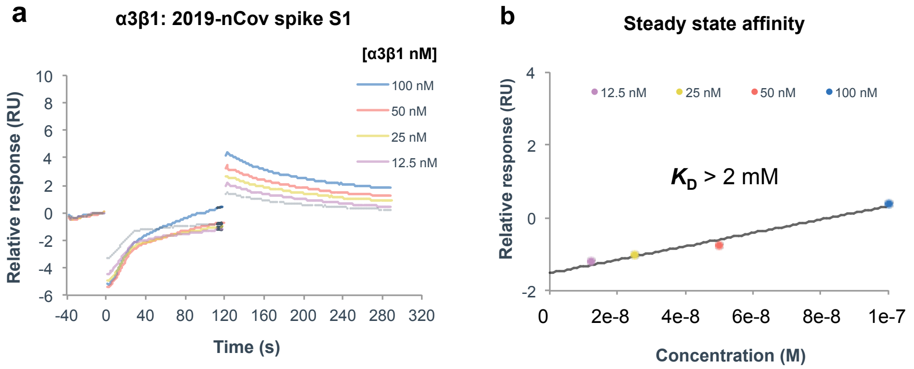

Supplementary Fig 8. 2019-nCov spike S1 protein did not bind α3β1 integrin in surface plasmon resonance assays. a, b 2019-nCov S1 protein was immobilized onto a CM5 sensor chip with α3β1 integrin applied as an analyte in a serial of increasing concentrations. As shown above, unlike αvβ3 integrin, α3β1 integrin serving as a binding control did not bind 2019-nCov-2 spike S1 protein well (**a**) with K_D>2 mM (**b**).

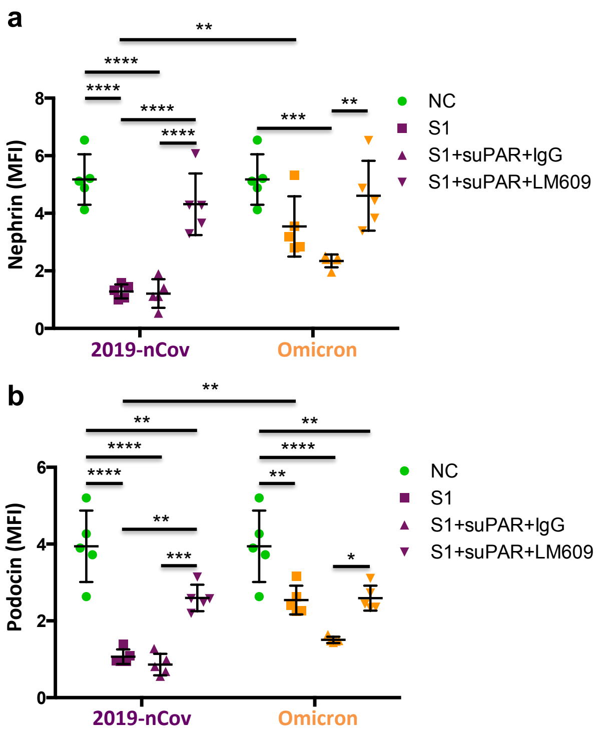

Supplementary Fig 9. 2019-nCov S1 protein elicited reduction in nephrin and podocin that was reversed by αvβ3 integrin blocking. Fully differentiated human podocytes were treated with 2019-nCov-2 S1 protein, suPAR and anti-αvβ3 integrin antibody LM609 for 16 hours before harvest for immunofluorescence staining and imaging. **a** Nephrin. **b** Podocin. MFI, mean fluorescence intensity. Data were presented as mean ± SD (n=5 biological replicates per group). Two-way ANOVA with Tukey's multiple comparisons test. NC, normal saline control. * *P*<0.05, ** *P*<0.01, *** *P*<0.001, **** *P*<0.0001.

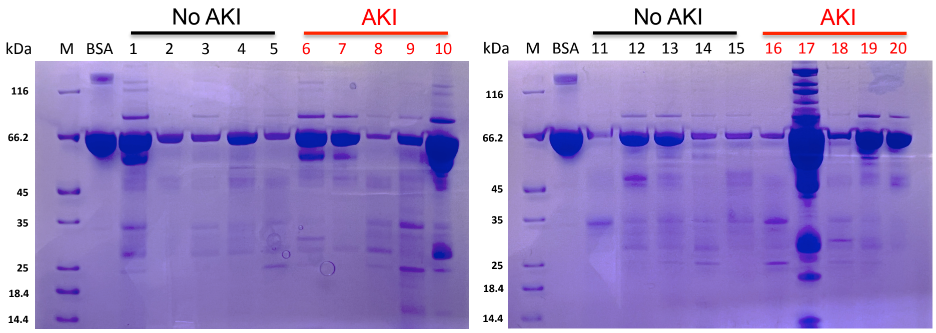

Supplementary Fig 10. Urine electrophoresis analyses with patients hospitalized for COVID-19. To determine the nature of proteinuria with COVID-19 patients, 20 patients (n=10 with clinical AKI, n=10 without clinical AKI) were randomly selected from M^2C^2 cohort. Equal amount of urine in total protein was loaded into NuPage Bis-Tris gel for separation, with BSA as positive control for albumin. The gels were then stained with Imperial protein stain for visualization. M, protein molecule weight marker. BSA, bovine serum albumin. 1-5, and 11-15, urine samples from COVID-19 without clinical AKI diagnosis. 6-10, and 16-20, urine samples from COVID-19 with clinical AKI diagnosis. AKI, acute kidney injury. The experiments were repeated twice with 20 independent samples, giving similar results.

Supplementary Table 1. Clinical characteristics of COVID-19 patients stratified by predominant variant

	Non-Omicron (n=1256)	Omicron (n=735)
Age	60 (17)	64 (19)
Male sex	710 (56.5%)	365 (49.7%)
Black race	303 (24.1%)	119 (16.2%)
Diabetes mellitus	530 (42.2%)	244 (33.2%)
Hypertension	780 (62.1%)	399 (54.3%)
Coronary artery disease	201 (16.0%)	143 (19.5%)
Heart failure	148 (11.8%)	110 (15.0%)
Estimated glomerular filtration rate on admission	68 (33)	68 (34)
Respiratory failure requiring mechanical ventilation	454 (36.1%)	111 (15.1%)
Proteinuria		
None	450 (35.8%)	334 (45.4%)
1+ (30-99 mg/dl)	463 (36.9%)	243 (33.1%)
2+ (100-300 mg/dl)	226 (18.0%)	98 (13.3%)
3+ (>300 mg/dl)	117 (9.3%)	60 (8.2%)

Supplementary Table 2. Omicron infections are independently associated with lower suPAR levels.

	suPAR (dependent variable)	
	β, 95%CI	*P*-value*
Age, per 1 year	-0.03 (-0.06--0.01)	0.001
Female vs. Male	0.31 (-0.28-0.90)	0.301
Black race vs. non-Black race	-1.19 (-1.88--0.50)	<0.001
Diabetes mellitus	0.29 (-0.34-0.92)	0.36
Hypertension	0.13 (-0.55-0.81)	0.70
Coronary artery disease	0.37 (-0.48-1.21)	0.40
Heart Failure	0.82 (-0.14-1.77)	0.10
Estimated glomerular filtration rate, per 1 ml/min/1.73 m^2	-0.05 (-0.06--0.04)	<0.001
Respiratory failure requiring mechanical ventilation	3.43 (2.81-4.06)	<0.001
Omicron vs. Non-omicron	-2.82 (-3.53--2.11)	<0.001

* linear regression, two-sided test, adjustments were made for multiple comparisons.

Supplementary Table 3. Omicron variants are associated with a lower odds of proteinuria.

	At least +1 proteinuria (dependent variable)	
	Odds ratio, 95%CI	*P*-value*
Age, per 1 year	1.01 (1.00-1.01)	0.12
Female vs. Male	0.68 (0.56-0.83)	<0.001
Black race vs. non-Black race	1.67 (1.30-2.15)	<0.001
Diabetes mellitus	1.22 (0.98-1.50)	0.07
Hypertension	0.97 (0.78-1.21)	0.78
Coronary artery disease	0.76 (0.58-1.00)	0.047
Heart Failure	0.82 (0.60-1.11)	0.19
Estimated glomerular filtration rate, per 1 ml/min/1.73 m^2	0.99 (0.98-0.99)	<0.001
Respiratory failure requiring mechanical ventilation	2.27 (1.80-2.86)	<0.001
Omicron vs. Non-omicron	0.82 (0.67-1.00)	0.048

* Binary logistic regression, two-sided test, adjustments were made for multiple comparisons.

Uncropped WB for Supplementary Fig. 7

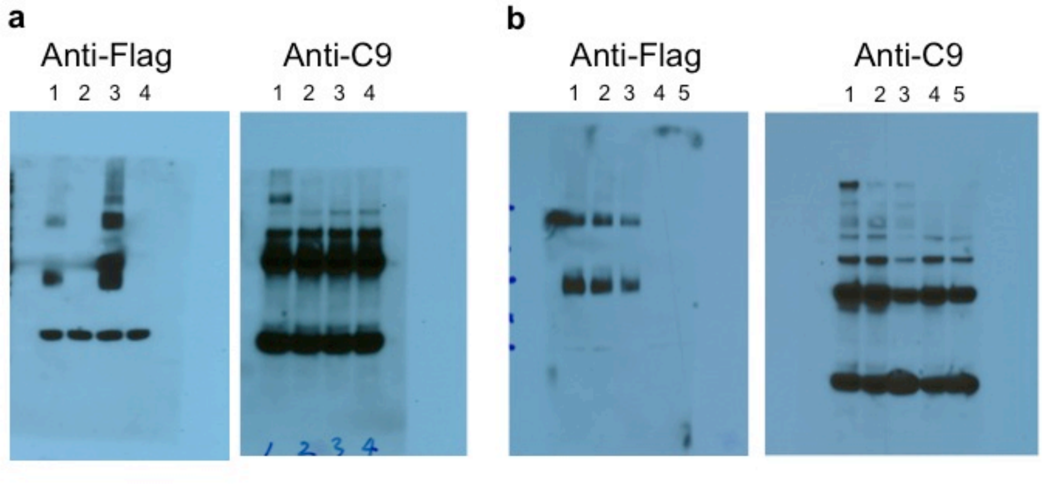

Uncropped gels for Supplementary Fig. 10

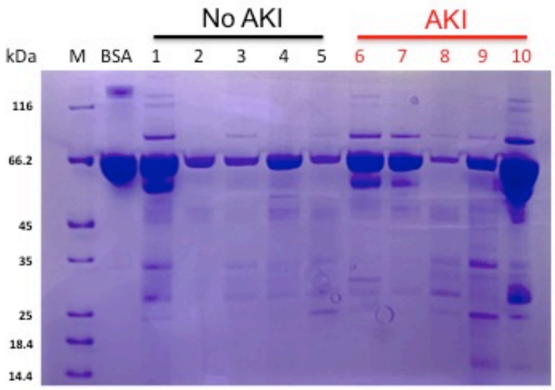

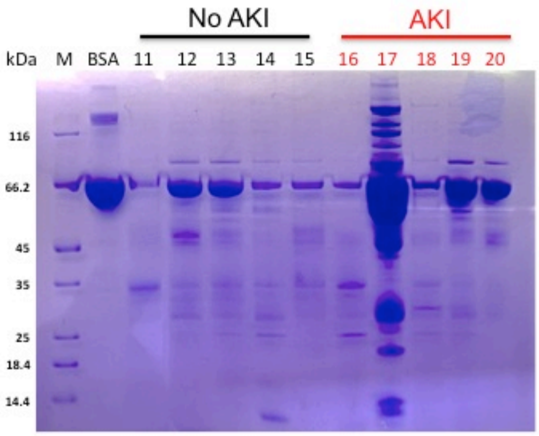

Supplement: Supplementary file 1 — Supplementary Information [file 41467_2023_40165_MOESM1_ESM.pdf]
