# Supplementary material for: SuPAR mediates viral response proteinuria by rapidly changing podocyte function
Source: Nat Commun. 2023 Jul 21;14:4414. doi: 10.1038/s41467-023-40165-5 (PMC10362037; doi:10.1038/s41467-023-40165-5)
Supplement: Supplementary file 3 — Reporting Summary [file 41467_2023_40165_MOESM3_ESM.pdf]

nature portfolio

Corresponding author(s): Jochen Reiser

Last updated by author(s): 06/21/2023

Reporting Summary

Nature Portfolio wishes to improve the reproducibility of the work that we publish. This form provides structure for consistency and transparency in reporting. For further information on Nature Portfolio policies, see our Editorial Policies and the Editorial Policy Checklist.

Statistics

For all statistical analyses, confirm that the following items are present in the figure legend, table legend, main text, or Methods section.

n/a	Confirmed	
☐	☒	The exact sample size (*n*) for each experimental group/condition, given as a discrete number and unit of measurement
☐	☒	A statement on whether measurements were taken from distinct samples or whether the same sample was measured repeatedly
☐	☒	The statistical test(s) used AND whether they are one- or two-sided<br>*Only common tests should be described solely by name; describe more complex techniques in the Methods section.*
☐	☒	A description of all covariates tested
☐	☒	A description of any assumptions or corrections, such as tests of normality and adjustment for multiple comparisons
☐	☒	A full description of the statistical parameters including central tendency (e.g. means) or other basic estimates (e.g. regression coefficient) AND variation (e.g. standard deviation) or associated estimates of uncertainty (e.g. confidence intervals)
☐	☒	For null hypothesis testing, the test statistic (e.g. *F*, *t*, *r*) with confidence intervals, effect sizes, degrees of freedom and *P* value noted<br>*Give P values as exact values whenever suitable.*
☒	☐	For Bayesian analysis, information on the choice of priors and Markov chain Monte Carlo settings
☒	☐	For hierarchical and complex designs, identification of the appropriate level for tests and full reporting of outcomes
☒	☐	Estimates of effect sizes (e.g. Cohen's *d*, Pearson's *r*), indicating how they were calculated

Our web collection on statistics for biologists contains articles on many of the points above.

Software and code

Policy information about availability of computer code

Data collection	NIS-Elements AR 5.30 (Nikon).
Data analysis	Graphpad Prism 6, SPSS statistics 27, R with ggplot2 package

For manuscripts utilizing custom algorithms or software that are central to the research but not yet described in published literature, software must be made available to editors and reviewers. We strongly encourage code deposition in a community repository (e.g. GitHub). See the Nature Portfolio guidelines for submitting code & software for further information.

Data

Policy information about availability of data

All manuscripts must include a data availability statement. This statement should provide the following information, where applicable:
- Accession codes, unique identifiers, or web links for publicly available datasets
- A description of any restrictions on data availability
- For clinical datasets or third party data, please ensure that the statement adheres to our policy

The authors declare that all data supporting the findings of this study are available within the paper, its supplementary and source data files.

Research involving human participants, their data, or biological material

Policy information about studies with human participants or human data. See also policy information about sex, gender (identity/presentation), and sexual orientation and race, ethnicity and racism.

Reporting on sex and gender	yes
Reporting on race, ethnicity, or other socially relevant groupings	yes
Population characteristics	yes
Recruitment	yes
Ethics oversight	yes

Note that full information on the approval of the study protocol must also be provided in the manuscript.

Field-specific reporting

Please select the one below that is the best fit for your research. If you are not sure, read the appropriate sections before making your selection.

☒ Life sciences ☐ Behavioural & social sciences ☐ Ecological, evolutionary & environmental sciences

For a reference copy of the document with all sections, see nature.com/documents/nr-reporting-summary-flat.pdf

Life sciences study design

All studies must disclose on these points even when the disclosure is negative.

Sample size	Sample size was determined by pilot study and Power analysis as well. Power analysis was performed with the aid of G*Power. 80% power was targeted, with type I error set as p=0.05. Two sided statistical tests were performed. In terms of effect size of proteinuria, the basal levels of untreated, healthy, age and gender matched C57BL/6j were used as reference. Any value more than the reference mean+2SD was considered effect size.
Data exclusions	no exclusions was made
Replication	Replication was attempted at different levels. 1. Whenever replication of whole experiment is not realistic, replication was made with different independent subjects. eg. African green monkey modeling. Replication of treatment was achieved with 4 independent monkeys in each group. Whenever replicating whole experiment is doable, eg cell culture, the experiments were repeated at least twice, i.e two different batches of cells were cultured before randomly split for different treatment. Within each batch of cells, replication of treatment was performed. All attempts of replication were achieved in this study.
Randomization	For animal studies, age-sex matched animals were numbered and randomly selected into either control or treatment groups. For cell based experiments, the same batch of cells were split into different aliquots, numbered and randomly selected to receive either treatment or vehicle control. For human subject, it was determined by inclusion/exclusion criteria specified in the study.
Blinding	Individual investigators were blinded, during group allocation for treatment and data collection/analysis.

Behavioural & social sciences study design

All studies must disclose on these points even when the disclosure is negative.

Study description	*Briefly describe the study type including whether data are quantitative, qualitative, or mixed-methods (e.g. qualitative cross-sectional, quantitative experimental, mixed-methods case study).*
Research sample	*State the research sample (e.g. Harvard university undergraduates, villagers in rural India) and provide relevant demographic information (e.g. age, sex) and indicate whether the sample is representative. Provide a rationale for the study sample chosen. For studies involving existing datasets, please describe the dataset and source.*
Sampling strategy	*Describe the sampling procedure (e.g. random, snowball, stratified, convenience). Describe the statistical methods that were used to predetermine sample size OR if no sample-size calculation was performed, describe how sample sizes were chosen and provide a rationale for why these sample sizes are sufficient. For qualitative data, please indicate whether data saturation was considered, and what criteria were used to decide that no further sampling was needed.*
Data collection	*Provide details about the data collection procedure, including the instruments or devices used to record the data (e.g. pen and paper, computer, eye tracker, video or audio equipment) whether anyone was present besides the participant(s) and the researcher, and*

	whether the researcher was blind to experimental condition and/or the study hypothesis during data collection.
Timing	*Indicate the start and stop dates of data collection. If there is a gap between collection periods, state the dates for each sample cohort.*
Data exclusions	*If no data were excluded from the analyses, state so OR if data were excluded, provide the exact number of exclusions and the rationale behind them, indicating whether exclusion criteria were pre-established.*
Non-participation	*State how many participants dropped out/declined participation and the reason(s) given OR provide response rate OR state that no participants dropped out/declined participation.*
Randomization	*If participants were not allocated into experimental groups, state so OR describe how participants were allocated to groups, and if allocation was not random, describe how covariates were controlled.*

Ecological, evolutionary & environmental sciences study design

All studies must disclose on these points even when the disclosure is negative.

Study description	*Briefly describe the study. For quantitative data include treatment factors and interactions, design structure (e.g. factorial, nested, hierarchical), nature and number of experimental units and replicates.*
Research sample	*Describe the research sample (e.g. a group of tagged Passer domesticus, all Stenocereus thurberi within Organ Pipe Cactus National Monument), and provide a rationale for the sample choice. When relevant, describe the organism taxa, source, sex, age range and any manipulations. State what population the sample is meant to represent when applicable. For studies involving existing datasets, describe the data and its source.*
Sampling strategy	*Note the sampling procedure. Describe the statistical methods that were used to predetermine sample size OR if no sample-size calculation was performed, describe how sample sizes were chosen and provide a rationale for why these sample sizes are sufficient.*
Data collection	*Describe the data collection procedure, including who recorded the data and how.*
Timing and spatial scale	*Indicate the start and stop dates of data collection, noting the frequency and periodicity of sampling and providing a rationale for these choices. If there is a gap between collection periods, state the dates for each sample cohort. Specify the spatial scale from which the data are taken*
Data exclusions	*If no data were excluded from the analyses, state so OR if data were excluded, describe the exclusions and the rationale behind them, indicating whether exclusion criteria were pre-established.*
Reproducibility	*Describe the measures taken to verify the reproducibility of experimental findings. For each experiment, note whether any attempts to repeat the experiment failed OR state that all attempts to repeat the experiment were successful.*
Randomization	*Describe how samples/organisms/participants were allocated into groups. If allocation was not random, describe how covariates were controlled. If this is not relevant to your study, explain why.*
Blinding	*Describe the extent of blinding used during data acquisition and analysis. If blinding was not possible, describe why OR explain why blinding was not relevant to your study.*

Did the study involve field work? ☐ Yes ☒ No

Field work, collection and transport

Field conditions	*Describe the study conditions for field work, providing relevant parameters (e.g. temperature, rainfall).*
Location	*State the location of the sampling or experiment, providing relevant parameters (e.g. latitude and longitude, elevation, water depth).*
Access & import/export	*Describe the efforts you have made to access habitats and to collect and import/export your samples in a responsible manner and in compliance with local, national and international laws, noting any permits that were obtained (give the name of the issuing authority, the date of issue, and any identifying information).*
Disturbance	*Describe any disturbance caused by the study and how it was minimized.*

Reporting for specific materials, systems and methods

We require information from authors about some types of materials, experimental systems and methods used in many studies. Here, indicate whether each material, system or method listed is relevant to your study. If you are not sure if a list item applies to your research, read the appropriate section before selecting a response.

Materials & experimental systems

n/a	Involved in the study	
☐	☒	Antibodies
☐	☒	Eukaryotic cell lines
☒	☐	Palaeontology and archaeology
☐	☒	Animals and other organisms
☐	☒	Clinical data
☒	☐	Dual use research of concern
☒	☐	Plants

Methods

n/a	Involved in the study	
☒	☐	ChIP-seq
☒	☐	Flow cytometry
☒	☐	MRI-based neuroimaging

Antibodies

Antibodies used	1. Rabbit anti-podocin, Immuno-Biological Laboratories Co LTD (IBL), #JP29040, Lot. 1F-826; 2. Mouse anti-alpha-Actinin-4, Santa Cruz, #sc-390205, G-4, Lot. A0521; 3. Rat anti-uPAR, R&D Systems, #MAB531, Clone 109801, Lot. EQJ0519112; 4. Rat IgG2a, R&D Systems, #MAB006, Lot. CAO2517081; 5. Mouse anti-V5, Invitrogen, #46-0705, Clone SV5-Pk1, Lot. 2418887; 6. Mouse anti-Myc, Sigma, #M4439, Clone 9E10, Lot. 026M4825V; 7. Mouse anti-HIS, Invitrogen, #MA1-21315, Clone HIS.H8, Lot. VB293165. 8. Mouse anti-Flag, Sigma, #F1804, Clone M2, Lot. 080M6035; 9. Goat anti-ACE2, R&D Systems, #AF933, Lot. HOK0320041; 10. Rabbit anti-podocin, Sigma, #P0372, Lot. 0000122935; 11. Mouse anti-beta3 integrin, Kerafast, #EBW107, Clone AP5; 12. Mouse anti-avb3 integrin, Millipore, #MAB1976, Clone LM609, Lot. LV1671288; 13. Mouse anti-uPAR, R&D Systems, #MAB807, Clone 62022, Lot. CEP0110071. 14. Mouse IgG1 isotype control, Invitrogen, #02-6100; 15. Mouse anti-nephrin (a gift from Dr. Karl Tryggvason); 16. Alpaca anti-rabbit IgG, recombinant VHH, Alexa Fluor 488, Chromotek, #srbAF488-1-100; 17. Cy™3 AffiniPure F(ab')$_2$ Fragment Goat Anti-Mouse IgG, F(ab')2 fragment specific, Jacksonimmuno, #115-166-006. 18. Alexa Fluor488 chicken anti-mouse IgG, Invitrogen, #A21200, Lot. 929536; 19. Alexa Fluor594 chicken anti-rabbit IgG, Invitrogen, #A21442, Lot. 2041636; 20. Alexa Fluor594 chicken anti-goat IgG, Invitrogen, #A21468, Lot. 877581. 21. C9 tag polyclonal antibody, Abnova, #PAB26959.
Validation	All primary antibodies have been validated by their supplying sources. 1. Anti-human podocin rabbit IgG, JP29040, IBL, cross-reacts with rat and mouse, recommended applications: IHC (frozen or FFPB sections), WB, IP. 2. alpha-actinin-4 (G4), sc-390205, Santa Cruz, recommended for detection of alpha-actinin-4 of mouse, rat, and human origin by WB, IP, IF and ELISA. 3. Rat anti-uPAR, MAB531, R&D Systems, detects mouse uPAR in Western blots. In Western blots, no cross-reactivity with recombinant human uPAR is observed. Recommended for WB, FC, blockade of receptor-ligand interaction. 4. Mouse anti-V5, 46-0705, Invitrogen, Species reactivity: Tag. Recommended use: WB, ICC/IF, ELISA, IP. 5. Mouse anti-HIS, MA1-21315, Invitrogen, Species reactivity: Tag, Recommended use: WB, ICC/IF, ELISA, IP. 6. Mouse anti-Myc, M4439, Sigma, Suitable for IP, WB, ELISA, IF, ChIP. 7. Mouse anti-Flag, F1804, Sigma, For highly sensitive and specific detection of FLAG fusion proteins by WB (IB), IP, IHC, IF, ICC. 8. Goat ant-human ACE2, AF933, R&D, Species reactivity: human, mouse, rat, hamster. Detects human ACE-2 in direct ELISAs. Detects human, mouse, and rat ACE-2 in WB. Detects Hamster ACE-2 in IHC. 9. Rabbit anti-podocin, P0372, Sigma, Recognizes human, mouse, and rat podocin. Has been used for WB and IHC. 10. Mouse anti-beta3 integrin, EBW107, Kerafast, Reactivity: human. Tested applications: WB, FC, IHC. 11. Mouse anti-avb3 integrin, MAB1976, Millipore, Species reactivity: Av, B, Ca, Ch, H, Mk, Po, Rb. Key applications: FC, IF, IHC, IP, FUNC. 12. Mouse anti-uPAR, MAB807, R&D, Species reactivity: Human. Applications: WB, FC, Blockade. 13. 13. C9 tag polyclonal antibody, PAB26959, Abnova, Specificity: Reacts with proteins which contain C9 sequence. Applications: ELISA, WB.

Eukaryotic cell lines

Policy information about cell lines and Sex and Gender in Research

Cell line source(s)	HEK293T was from ATCC; human podocytes were initially from Dr. Saleem Moin and maintained in our lab
Authentication	Authentication of HEK293T was achieved with the morphology, and the presence of Ad5 and SV40 large T antigen. Authentication of human podocytes was achieved through the morphology and the presence of podocyte marker proteins nephrin and podocin.
Mycoplasma contamination	tested negative
Commonly misidentified lines (See ICLAC register)	No commonly misidentified lines

Palaeontology and Archaeology

Specimen provenance	*Provide provenance information for specimens and describe permits that were obtained for the work (including the name of the issuing authority, the date of issue, and any identifying information). Permits should encompass collection and, where applicable, export.*
Specimen deposition	*Indicate where the specimens have been deposited to permit free access by other researchers.*

Dating methods | *If new dates are provided, describe how they were obtained (e.g. collection, storage, sample pretreatment and measurement), where they were obtained (i.e. lab name), the calibration program and the protocol for quality assurance OR state that no new dates are provided.*

☐ Tick this box to confirm that the raw and calibrated dates are available in the paper or in Supplementary Information.

Ethics oversight | *Identify the organization(s) that approved or provided guidance on the study protocol, OR state that no ethical approval or guidance was required and explain why not.*

Note that full information on the approval of the study protocol must also be provided in the manuscript.

Animals and other research organisms

Policy information about studies involving animals; ARRIVE guidelines recommended for reporting animal research, and Sex and Gender in Research

Laboratory animals	C57BL/6j mice (10-12 weeks old), uPAR knockout mice (10-12 weeks old), suPAR transgenic mice (10-12 weeks old) and IL-5 transgenic mice (10-12 weeks old); African Green Moneys (16 years old).
Wild animals	no wild animals were used.
Reporting on sex	Both male and female animals were used overall, except that in SARS-Cov-2 spike S1 protein or nucleocapsid protein inhalation models, only male mice were used per protocol.
Field-collected samples	no field-collected samples were used.
Ethics oversight	For non-human primate African green monkeys, The Institutional Animal Care and Use Committee of Tulane University reviewed and approved [Nonhuman Primate (NHP)] Model Development for SARS CoV-2 Infection, #P0435) and all the procedures for this study. All mouse work in this study was approved and overseen by Rush University Institutional Animal Care and Use Committee under protocol #22-019.

Note that full information on the approval of the study protocol must also be provided in the manuscript.

Clinical data

Policy information about clinical studies

All manuscripts should comply with the ICMJEguidelines for publication of clinical research and a completedCONSORT checklist must be included with all submissions.

Clinical trial registration	n/a
Study protocol	yes
Data collection	yes
Outcomes	yes

Dual use research of concern

Policy information about dual use research of concern

Hazards

Could the accidental, deliberate or reckless misuse of agents or technologies generated in the work, or the application of information presented in the manuscript, pose a threat to:

No	Yes	
☒	☐	Public health
☒	☐	National security
☒	☐	Crops and/or livestock
☒	☐	Ecosystems
☒	☐	Any other significant area

Experiments of concern

Does the work involve any of these experiments of concern:

No	Yes	
☒	☐	Demonstrate how to render a vaccine ineffective
☒	☐	Confer resistance to therapeutically useful antibiotics or antiviral agents
☒	☐	Enhance the virulence of a pathogen or render a nonpathogen virulent
☒	☐	Increase transmissibility of a pathogen
☒	☐	Alter the host range of a pathogen
☒	☐	Enable evasion of diagnostic/detection modalities
☒	☐	Enable the weaponization of a biological agent or toxin
☒	☐	Any other potentially harmful combination of experiments and agents

Plants

Seed stocks

Report on the source of all seed stocks or other plant material used. If applicable, state the seed stock centre and catalogue number. If plant specimens were collected from the field, describe the collection location, date and sampling procedures.

Novel plant genotypes

Describe the methods by which all novel plant genotypes were produced. This includes those generated by transgenic approaches, gene editing, chemical/radiation-based mutagenesis and hybridization. For transgenic lines, describe the transformation method, the number of independent lines analyzed and the generation upon which experiments were performed. For gene-edited lines, describe the editor used, the endogenous sequence targeted for editing, the targeting guide RNA sequence (if applicable) and how the editor was applied.

Authentication

Describe any authentication procedures for each seed stock used or novel genotype generated. Describe any experiments used to assess the effect of a mutation and, where applicable, how potential secondary effects (e.g. second site T-DNA insertions, mosiacism, off-target gene editing) were examined.

ChIP-seq

Data deposition

☐ Confirm that both raw and final processed data have been deposited in a public database such as GEO.

☐ Confirm that you have deposited or provided access to graph files (e.g. BED files) for the called peaks.

Data access links
May remain private before publication.

For "Initial submission" or "Revised version" documents, provide reviewer access links. For your "Final submission" document, provide a link to the deposited data.

Files in database submission

Provide a list of all files available in the database submission.

Genome browser session
(e.g. UCSC)

Provide a link to an anonymized genome browser session for "Initial submission" and "Revised version" documents only, to enable peer review. Write "no longer applicable" for "Final submission" documents.

Methodology

Replicates

Describe the experimental replicates, specifying number, type and replicate agreement.

Sequencing depth

Describe the sequencing depth for each experiment, providing the total number of reads, uniquely mapped reads, length of reads and whether they were paired- or single-end.

Antibodies

Describe the antibodies used for the ChIP-seq experiments; as applicable, provide supplier name, catalog number, clone name, and lot number.

Peak calling parameters

Specify the command line program and parameters used for read mapping and peak calling, including the ChIP, control and index files used.

Data quality

Describe the methods used to ensure data quality in full detail, including how many peaks are at FDR 5% and above 5-fold enrichment.

Software

Describe the software used to collect and analyze the ChIP-seq data. For custom code that has been deposited into a community repository, provide accession details.

Flow Cytometry

Plots

Confirm that:

- [ ] The axis labels state the marker and fluorochrome used (e.g. CD4-FITC).
- [ ] The axis scales are clearly visible. Include numbers along axes only for bottom left plot of group (a 'group' is an analysis of identical markers).
- [ ] All plots are contour plots with outliers or pseudocolor plots.
- [ ] A numerical value for number of cells or percentage (with statistics) is provided.

Methodology

Sample preparation	*Describe the sample preparation, detailing the biological source of the cells and any tissue processing steps used.*
Instrument	*Identify the instrument used for data collection, specifying make and model number.*
Software	*Describe the software used to collect and analyze the flow cytometry data. For custom code that has been deposited into a community repository, provide accession details.*
Cell population abundance	*Describe the abundance of the relevant cell populations within post-sort fractions, providing details on the purity of the samples and how it was determined.*
Gating strategy	*Describe the gating strategy used for all relevant experiments, specifying the preliminary FSC/SSC gates of the starting cell population, indicating where boundaries between "positive" and "negative" staining cell populations are defined.*

- [ ] Tick this box to confirm that a figure exemplifying the gating strategy is provided in the Supplementary Information.

Magnetic resonance imaging

Experimental design

Design type	*Indicate task or resting state; event-related or block design.*
Design specifications	*Specify the number of blocks, trials or experimental units per session and/or subject, and specify the length of each trial or block (if trials are blocked) and interval between trials.*
Behavioral performance measures	*State number and/or type of variables recorded (e.g. correct button press, response time) and what statistics were used to establish that the subjects were performing the task as expected (e.g. mean, range, and/or standard deviation across subjects).*

Acquisition

Imaging type(s)	*Specify: functional, structural, diffusion, perfusion.*
Field strength	*Specify in Tesla*
Sequence & imaging parameters	*Specify the pulse sequence type (gradient echo, spin echo, etc.), imaging type (EPI, spiral, etc.), field of view, matrix size, slice thickness, orientation and TE/TR/flip angle.*
Area of acquisition	*State whether a whole brain scan was used OR define the area of acquisition, describing how the region was determined.*

Diffusion MRI ☐ Used ☐ Not used

Preprocessing

Preprocessing software	*Provide detail on software version and revision number and on specific parameters (model/functions, brain extraction, segmentation, smoothing kernel size, etc.).*
Normalization	*If data were normalized/standardized, describe the approach(es): specify linear or non-linear and define image types used for transformation OR indicate that data were not normalized and explain rationale for lack of normalization.*
Normalization template	*Describe the template used for normalization/transformation, specifying subject space or group standardized space (e.g. original Talairach, MNI305, ICBM152) OR indicate that the data were not normalized.*
Noise and artifact removal	*Describe your procedure(s) for artifact and structured noise removal, specifying motion parameters, tissue signals and physiological signals (heart rate, respiration).*

Volume censoring | *Define your software and/or method and criteria for volume censoring, and state the extent of such censoring.*

Statistical modeling & inference

Model type and settings | *Specify type (mass univariate, multivariate, RSA, predictive, etc.) and describe essential details of the model at the first and second levels (e.g. fixed, random or mixed effects; drift or auto-correlation).*

Effect(s) tested | *Define precise effect in terms of the task or stimulus conditions instead of psychological concepts and indicate whether ANOVA or factorial designs were used.*

Specify type of analysis: ☐ Whole brain ☐ ROI-based ☐ Both

Statistic type for inference | *Specify voxel-wise or cluster-wise and report all relevant parameters for cluster-wise methods.*

(See Eklund et al. 2016)

Correction | *Describe the type of correction and how it is obtained for multiple comparisons (e.g. FWE, FDR, permutation or Monte Carlo).*

Models & analysis

n/a	Involved in the study
☐	☐ Functional and/or effective connectivity
☐	☐ Graph analysis
☐	☐ Multivariate modeling or predictive analysis

Functional and/or effective connectivity | *Report the measures of dependence used and the model details (e.g. Pearson correlation, partial correlation, mutual information).*

Graph analysis | *Report the dependent variable and connectivity measure, specifying weighted graph or binarized graph, subject- or group-level, and the global and/or node summaries used (e.g. clustering coefficient, efficiency, etc.).*

Multivariate modeling and predictive analysis | *Specify independent variables, features extraction and dimension reduction, model, training and evaluation metrics.*
